# Commentary: Ultrasound-guided acupuncture therapy in Korea: advancing traditional practices with new technology

**DOI:** 10.3389/fmed.2025.1603302

**Published:** 2025-05-14

**Authors:** Zhongtian Chen, Shangdong Li, Renyu Yang, Ruimin Liu

**Affiliations:** ^1^First Clinical Medical College, Gansu University of Chinese Medicine, Lanzhou, China; ^2^Gansu Provincial Hospital, Lanzhou, China

**Keywords:** ultrasound-guided, acupuncture, Korea medicine, traditional medical doctors, clinical guideline

We write to expand upon the compelling work by Lee et al. titled “Ultrasound-guided acupuncture therapy in Korea: advancing traditional practices with new technology” (1), which highlights the growing role of ultrasound-guided acupuncture (USGA) in Korean Medicine (KM). As a multidisciplinary team of surgeons, pain specialists, and researchers, we applaud the authors for bridging traditional acupuncture with modern imaging technologies—a synergy with profound implications for surgical and perioperative care. However, to fully realize the potential of USGA in contemporary medicine, critical challenges must be addressed through rigorous research, policy reform, and cross-disciplinary collaboration.

## USGA as a surgical adjuvant: precision beyond conventional needling

The integration of ultrasonography into acupuncture practice, as detailed by Lee et al., offers unprecedented precision in targeting anatomical structures such as the subacromial-subdeltoid bursa or peripheral nerves. This advancement holds particular promise for surgical applications, where minimally invasive techniques increasingly dominate clinical practice. For instance, USGA could augment postoperative rehabilitation by delivering pharmacopuncture agents directly to injured tendons or ligaments under real-time imaging, potentially reducing reliance on systemic analgesics (2). In thoracic or abdominal surgeries, where inadvertent needling risks pneumothorax or visceral injury, USGA's ability to visualize tissue planes may mitigate complications. Yet, to validate its surgical utility, comparative trials are urgently needed—for example, comparing USGA-assisted trigger point needling against ultrasound-guided corticosteroid injections in rotator cuff repair patients. Such studies should prioritize endpoints like functional recovery timelines and opioid consumption, aligning with surgical outcome frameworks.

## Training surgeons and Korean medicine doctors: a shared responsibility

The technical demands of USGA—proficiency in sonographic anatomy, needle navigation, and dynamic imaging interpretation—mirror those required for ultrasound-guided surgical procedures, such as nerve blocks or tumor ablations. However, Lee et al. note that KM doctors in Korea face a steep learning curve due to fragmented training opportunities. We propose that surgical and KM training programs collaborate to develop hybrid curricula. For example, simulation-based modules used in surgical residencies—e.g., virtual reality platforms for ultrasound-guided biopsies—could be adapted for USGA education. Joint workshops led by surgeons and KM experts would foster mutual understanding of anatomical landmarks and procedural safety, particularly for high-risk regions like the thoracic spine (3). Notably, validated surgical simulation platforms could be repurposed for USGA education with procedural adaptations: SonoSim^®^ Ultrasound Training Solution (4): This modular system, employed in surgical residency for real-time needle guidance during central venous access, could be reconfigured with KM-specific modules. BodyViz^®^ 3D Anatomy Software (5): A VR platform reconstructing CT/MRI data into interactive haptic models, this tool enables safe exploration of complex needle trajectories in deep tissues (e.g., gluteal or subscapular regions). Vimedix^®^ CAE Healthcare: A high-fidelity simulator with adjustable tissue layers and complication scenarios (e.g., simulated vascular puncture), this system has demonstrated efficacy in Korean surgical biopsy training (6).

## Policy reform: aligning USGA with global surgical standards

The exclusion of USGA from South Korea's national health insurance, as highlighted by the authors, not only limits patient access but also stifles innovation in surgical adjunctive therapies. In contrast, countries like Germany and China have incorporated similar techniques (e.g., ultrasound-guided dry needling for myofascial pain) into insurance frameworks following robust cost-effectiveness analyses. For USGA to gain parity, we urge Korean health authorities to commission studies evaluating its long-term economic impact—e.g., reduced hospital readmissions for chronic pain or shorter postoperative recovery periods. Concurrently, professional societies representing both surgeons and Korean medicine doctors should jointly advocate for procedural reimbursement, leveraging precedents such as the 2022 Supreme Court ruling that legitimized KM doctors' use of ultrasonography.

## Synergies with surgical innovation

While Lee et al. focus on USGA's standalone applications, its integration with cutting-edge surgical technologies remains unexplored. For instance, combining USGA with platelet-rich plasma (PRP) or mesenchymal stem cell injections could enhance regenerative outcomes in tendon repair (7). Similarly, robotic needle placement systems, now routine in prostate biopsies, could be adapted for USGA to improve reproducibility in deep-tissue acupuncture. Artificial intelligence (AI) also presents opportunities: machine learning algorithms trained on datasets could predict optimal needle trajectories or automate adverse event detection, akin to AI-driven ultrasound tools in obstetrics (8).

## Ethical imperatives: equity and interprofessional trust

Historical tensions between KM and Western medicine in Korea, exemplified by legal disputes over ultrasound use, risk perpetuating silos that hinder patient care. As surgeons, we recognize that USGA's success depends on dismantling these barriers through ethical collaboration. This includes transparent reporting of adverse events (e.g., pneumothorax rates in thoracic USGA) and shared decision-making frameworks that empower patients to choose between USGA and conventional surgical options (9). Within this context, the strategic enhancement of the Korea Adverse Event Reporting System (KAERS) assumes particular significance. As a national pharmacovigilance platform currently focused on herbal medicine safety surveillance, expanding its mandate to include USGA-specific complications (e.g., pneumothorax, organ perforation) would achieve dual objectives: alignment with the legislative intent of the 2023 Korean Medicine Safety Act amendments emphasizing full-cycle supervision of medical technologies, and provision of standardized datasets crucial for cross-disciplinary safety evaluations. Such systemic evolution would establish KAERS as an evidence-based cornerstone for reconciling traditional and modern medical paradigms through robust adverse event documentation.

Furthermore, global health equity must be prioritized: if USGA proves effective, low-resource regions lacking surgical infrastructure could adopt it as a low-cost alternative for chronic pain management, contingent on international knowledge transfer.

## A roadmap for cross-disciplinary leadership

**Figure 1:**
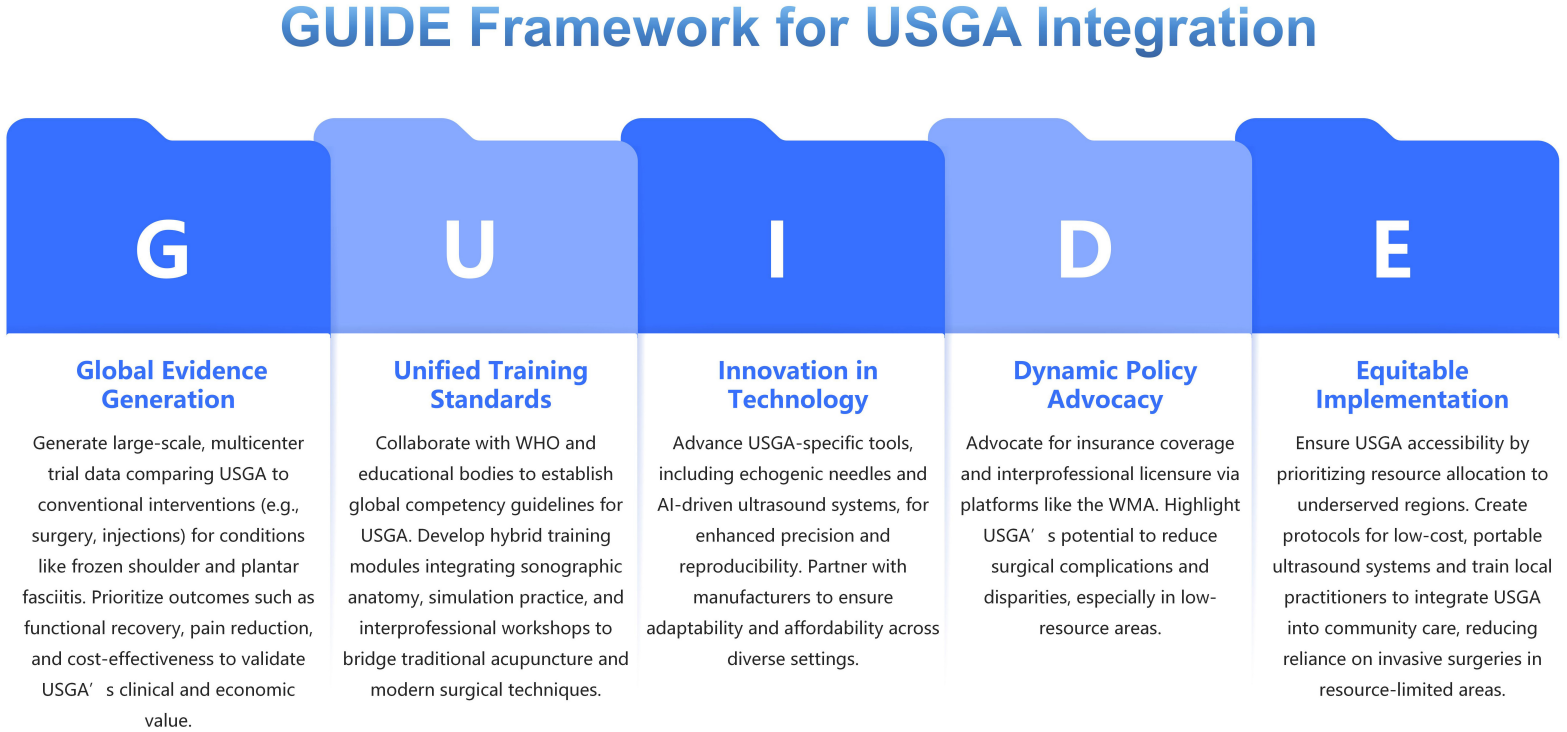
GUIDE framework for USGA integration.

To propel USGA from niche practice to mainstream surgical adjunct, we propose the following actions: GUIDE Framework for USGA Integration (Figure 1).

## G: Global evidence generation

Conduct large-scale, multicenter trials comparing ultrasound-guided acupuncture (USGA) to conventional surgical and non-surgical interventions (e.g., arthroscopic procedures, corticosteroid injections) for conditions like frozen shoulder and plantar fasciitis. Prioritize endpoints such as functional recovery, pain reduction, and cost-effectiveness to establish USGA's clinical and economic value.

## U: Unified training standards

Develop global competency guidelines for USGA through partnerships with the WHO and surgical/KM educational bodies. Integrate hybrid training modules—combining sonographic anatomy, simulation-based practice, and interprofessional workshops—to bridge gaps between traditional acupuncture and modern surgical techniques.

## I: Innovation in technology

Accelerate the design of USGA-specific tools, including echogenic needles and AI-driven ultrasound systems, to enhance precision and reproducibility. Collaborate with medical device manufacturers to ensure affordability and adaptability across diverse clinical settings.

## D: Dynamic policy advocacy

Lobby for insurance reimbursement and interprofessional licensure agreements, leveraging platforms like the World Medical Association. Highlight USGA's potential to reduce surgical complications and healthcare disparities, particularly in low-resource regions.

## E: Equitable implementation

Ensure USGA's accessibility across socioeconomic and geographic divides by prioritizing resource allocation to underserved areas. Develop protocols for low-cost, portable ultrasound systems and train local practitioners to integrate USGA into community care, reducing reliance on invasive surgeries in regions with limited healthcare infrastructure.

Lee et al.'s work marks a pivotal step toward harmonizing tradition and innovation. By addressing these challenges through surgical-KM collaboration, South Korea can emerge as a global exemplar of integrative medicine.
